# Heterodimeric IL-15 regulates the differentiation and survival of different populations of memory T cells and the balance of effector and regulatory cells

**DOI:** 10.1186/2051-1426-1-S1-P109

**Published:** 2013-11-07

**Authors:** George N Pavlakis, Cristina Bergamaschi, Jinyao Li, Antonio Valentin, Stephanie Chen, Sinnie S Ng, Rachel E Kelly Beach, Jenifer Bear, Margherita Rosati, Candido Alicea, Raymond Sowder, Elena Chertova, Barbara K Felber

**Affiliations:** 1 CCR, National Cancer Institute, Frederick, MD, USA

## 

The common γ-chain cytokine interleukin-15 (IL-15) regulates immune homeostasis and the fate of many lymphocyte subsets, and holds potential in fighting infections and cancer. We have previously showed that co-expression of IL-15 and IL-15 Receptor alpha (IL-15Rα) in the same cell allows for efficient production and secretion of bioactive IL-15/IL-15Rα heterodimer, whereas single-chain IL-15 is unstable. This led to the hypothesis that the physiologically relevant molecule in vivo is the heterodimer. Consistent with this hypothesis, we determined that the IL-15 found in the plasma of mice and humans is the heterodimer. Repeated subcutaneous administration of purified IL-15 heterodimers in macaques and mice resulted in sustained plasma IL-15 levels and in dose-dependent expansion of NK and T cells in blood and tissues, demonstrating pharmacokinetics and in vivo bioactivity superior to monomer IL-15. Even at a dose of 50 µg/Kg, the cytokine was well tolerated by macaques with no major side effects. IL-15 heterodimer promotes the preferential expansion of CD8+NK and CD8+ and CD4+ effector T (Teffs) cells, without preferentially affecting Tregs. As a result, sustained IL-15 levels are associated with lower relative frequency of Tregs and an increased ratio of Teff/Tregs in lymphoid tissues and in transplanted mouse colon carcinoma tumors. IL-15 knock-out (KO) mice were used to evaluate the contribution of IL-15 in the regulation of general and vaccine-induced memory T cells. Both CD4 and CD8 CD44hi Tem cells accumulate in IL-15 KO mice, consistent with the hypothesis that IL-15 is involved in the transition of effector cells to long-term central memory pool. Similar to the total Tem population, vaccine-induced Tem cells persist for longer periods of time in the absence of IL-15, providing evidence of a block in transition to long-term memory. In the absence of IL-15 Tcm cells are not sustained and there is evidence of increased apoptosis of these cells. These experiments provide information on the diverse effects of Heterodimeric IL-15 on different subpopulations of lymphocytes and support the hypothesis that IL-15 is required at multiple steps of memory T cell life cycle for the survival or differentiation of specific cell populations.

